# Biomimetic Scaffold of Chitosan from *Litopenaeus vannamei* Shrimp Shells Incorporated with Collagen and Hydroxyapatite for Bone Tissue Regeneration

**DOI:** 10.34172/apb.025.45909

**Published:** 2025-12-21

**Authors:** Cheryn Ivana, Hendrik Satria Dwi Putra, Nyoman Bayu Wisnu Kencana, Agustina Setiawati

**Affiliations:** Department of Pharmaceutical Biology, Faculty of Pharmacy, Sanata Dharma University, Yogyakarta, Indonesia

**Keywords:** Polymer, Shrimp shells, *Litopenaeus vannamei*, Bone graft, Tissue regeneration

## Abstract

**Introduction:**

The goal of this study was to create a biocompatible bone tissue engineering scaffold from *Litopenaeus vannamei* shrimp shell chitosan with hydroxyapatite (HAP) and collagen type I (COL I), and to investigate its physicochemical and biological properties relative to commercial chitosan scaffolds.

**Methods:**

The chitosan was extracted by following steps: deproteination, demineralization, and deacetylation processes, further characterizing it through composite FTIR spectroscopy. The scaffolds were fabricated using lyophilization, followed by the evaluation of their morphology, porosity, swelling ratio, degradation rate, and compressive strength. The scaffold morphology and porosity were observed using Scanning Electron Microscopy (SEM) and the solvent displacement method. Swelling and degradation ratio were investigated in phosphate buffer saline, while compressive strength was investigated with the Universal Testing Method. For the assessment of cytocompatibility, MG-63 osteoblast-like cells were subjected to the MTT assay, and cell morphology on the scaffold was observed under SEM.

**Results:**

As shown in the FTIR results, the derived chitosan exhibited comparable functional groups to those of commercial chitosan, confirming its successful extraction. Both scaffolds exhibited interconnected pores and suitable diameters and porosity for bone scaffold tissue engineering. Chitosan from shrimp shells showed a reduction in swelling (278.55±36.49% at 24h) and a slower degradation rate (41.87±7.27% at 4 weeks) compared to commercial scaffolds, possibly due to higher residual minerals and a lower degree of deacetylation. However, compressive strength 0.991±0.01 MPa and attachment and proliferation of MG-63 cells were similar, suggesting good osteoconductivity of the biomaterials.

**Conclusion:**

Chitosan-derived shrimp shells are a sustainable biomaterial candidate for bone tissue engineering. The scaffold based on shrimp shells exhibited relatively lower degradation and moderate swelling, adequate mechanical stability, and bioactivity to support osteoblast-like cell adhesion and viability, suggesting it is adequate for bone tissue engineering applications.

## Introduction

 Bone is a complex connective tissue that provides mechanical support, blood production, and organ protection, which is remarkably dynamic, continuously self-regenerates, and repairs after damage.^1^ For instance, in the case of tiny bone fractures, simple immobilization allows spontaneous healing over time. Defects caused by fracture, trauma, tumor excision, spinal deformity, and infection may surpass the critical size threshold and fail to heal natural physiological mechanism, thereby necessitating the use of supplementary materials to bridge the gap.^2-3^ The healing of bone defects has progressed through medical guidelines for bone grafting, including autografts and allografts, which are high-cost and high-infection-risk.^4-5^ Bone tissue engineering (BTE) is a potential alternative for autografts and allografts, employing synthetic grafts to promote tissue regeneration. An artificial graft, a scaffold, should serve as a filler within the defect site and encourage bone regrowth.^1^ To facilitate bone regeneration, a scaffold should be osteoconductive, enabling bone growth on its surface, while also providing a highly porous framework.^6,7^ In addition to these characteristics, a scaffold should have sufficient mechanical strength to sustain bone ingrowth at the implantation site, preserve structural integrity throughout *in vivo* tissue remodeling, and degrade over time in tandem with bone regeneration.^9,8^

 Both natural and synthetic polymers have been widely explored for scaffold fabrication.^9^ Among them, chitosan, a deacetylated form of chitin, is the primary structural biopolymer in crustacean exoskeletons such as shrimps, lobsters, and crabs.^10^ It has gained particular attention and featured a linear structure consisting of β(1-4)glycosidic bonds connecting d-glucosamine residues, interspersed with varying numbers of randomly distributed N-acetyl-d-glucosamine (NAG) units.^1^ It serves as a good substrate for cell adhesion and proliferation to secrete extracellular matrix (ECM), which mimics the natural structural protein of bone ECM. Thus, it serves to deliver growth factors to improve bone regeneration by providing a biomimetic microenvironment for bone regeneration.^11^ Due to its biodegradability, biocompatibility, antibacterial effect, and porous structure-forming ability, chitosan is considered a highly versatile biomaterial for tissue engineering.^6,12,13^ Our previous studies successfully engineered chitosan from mangrove and blue swimming crab to be a biomimetic composite for bone tissue engineering.^14,15^

 Thus, this study uses collagen, chitosan from shrimp (*Litopenaeus vannamei*) as a sustainable resource, aligning with United Nations Sustainable Development Goals (SDGs) by following deproteination, demineralization, and deacetylation steps.^16,17^ The shrimp shells contained more chitosan with a higher degree of deacetylation than that extracted from blue swimming crab and mangrove shells in our previous study.^13,14,17,18^ Since the human bone consists of a majority of collagen I (COL I) and hydroxyapatite (HAP), this study combined shrimp shells-derived chitosan into a scaffold to improve biocompatibility with tissue and impede toxicity effects. Moreover, COL I owes arginine-glycine-aspartic acid (RGD) peptides, which are a domain for α1β1 and α2β1 integrin receptors of the cells.^19,20^ The application of tissue-specific ECM components is an excellent strategy to grow specific cells ^21^ in bone tissue engineering. COL I on the scaffold provides a chemical biomimetic component since it is the major ECM component on human bone,^22-23^; however, the composition needs further development by combining with HAP, which structurally supports bone, leading to ectopic bone formation.^24-25^ HAP is an apatite mineral that occurs naturally. It makes up 70% of bone; therefore, it stimulates osteoblast proliferation independent of the extracellular matrix and facilitates osteointegration. HAP not only improves the scaffold strength and osteoconductivity, but also prevents the scaffold from defects.^26,27,28^ Previous studies have also highlighted the synergistic effects of combining chitosan with COL I and HAP for bone tissue engineering. Chitosan, COL I, and HAP scaffolds have been shown to enhance cellular adhesion and proliferation, as well as promote osteoblast and stem cell differentiation.^27,29,30^ The combination of COL I and HAP significantly upregulated osteogenic markers compared to chitosan alone.^31^ Our results highlight the potential of shrimp shell–derived chitosan enriched with COL I and HAP as an innovative biomaterial for bone tissue regeneration.

 In this study, the main goal is to develop sustainable bone tissue engineering based on chitosan isolated from shrimp (*Litopenaeus vannamei*) shells. The extracted chitosan was characterized structurally and chemically, then combined with HAP and COL I into a three-dimensional scaffold that structurally and compositionally mimics the natural bone. The composite scaffold was engineered through simple lyophilization without using a crosslinker. Despite having discovered that other researchers have also investigated chitosan-based composite scaffolds for bone tissue engineering, their exceptional performance and the use of shrimp shells as a chitosan source and a straightforward production process, absent of cross-linking, have never been published.

## Materials and Methods

###  Materials

*Litopenaeus vannamei* was collected from a local market in Yogyakarta in January 2023 and was authenticated in the Laboratory of Animal Systematics, Faculty of Biology, Universitas Gadjah Mada (N.46/BI/SH/III/2023). Sigma-Aldrich supplied commercial chitosan (419419), Pluronic F127 (P2443), bovine skin collagen (C4243), and paraformaldehyde (15817). Gibco also provided phosphate-buffered saline (10010-023). Phosphoric acid was supplied by Merck (1.000573.1000). The number of living cells was measured with Invitrogen’s 3-(4,5-dimethylthiazol-2-yl)-2,5-diphenyltetrazolium bromide (MTT) (M6494). For extraction, sodium hydroxide (NaOH) (1310-73-2) was purchased from Topaz Chemical, and hydrochloric acid was purchased from Smart Lab (100317). MG63 cells were propagated in high glucose medium Dulbecco’s Modified Eagle’s Medium (DMEM, Gibco) containing 10% Fetal Bovine Serum (FBS, Gibco 16000044) and 1% penicillin-streptomycin (Gibco, 10378016). All the well plates, pipettes, and tips used in this work were obtained from SPL Life Science, Corning, and Biologix.

###  Chitosan Extraction and Characterization

 The shells were removed, sorted, and cleaned using flowing water. The shrimp shells were dried in the oven, powdered, and stored in an air-tight container until used. The chitosan extraction from shrimp shells was conducted according to our previous studies with deproteination, demineralization, and deacetylation.^13,14^ The isolated chitosan was then confirmed by FTIR spectroscopy to detect its functional group compared to the commercially available chitosan.

###  Determination of Degree of Deacetylation (DD) of Extracted Chitosan

 This study estimated the degree of deacetylation (DD) of shrimp shell chitosan and commercial chitosan using FTIR spectroscopy, specifically based on the absorption bands observed at 1320 and 1420 cm^-1^.^32^ The band at 1320 cm^-1^ is attributed to the acetylated amine or amide functionality, whereas the band at 1420 cm^-1^ has been validated in previous studies as a reliable reference for DD determination, in agreement with ¹H NMR and ¹³C NMR studies reported by Czechowska-Biskup et al.^33^ The DD values were subsequently calculated based on a previous study using the subsequent equation^32^:


$$DD\%=100-\left(\frac{A1320}{A1420}-0.3822\right)*\frac{1}{0.03133}$$

###  Scaffold Fabrication

 To prepare the composite, hydroxyapatite (1.75 g) was first dissolved in 2% phosphoric acid and stirred by a magnetic stirrer. Chitosan (0.5 g), dissolved in 10 mL of acetic acid, was then incorporated into the hydroxyapatite solution to form a mixture. The composite was then mixed with bovine collagen (0.25 g) solution in acetic acid (10 mL). The scaffold solution was then neutralized to pH 7 with sodium hydroxide and adjusted with demineralized water until it contained HAP, chitosan, and collagen at final concentrations of 175, 50, and 25 μg/mL, precisely. The prepared solution was cast into a silicon mold and frozen at −80 °C for 24 hours to preserve its structure, followed by lyophilization for 24 hours to remove residual solvents and obtain a porous composite. A control scaffold was also engineered using medium-molecular-weight chitosan that was bought commercially, applying the same methods and materials as the chitosan that was isolated from the shrimp shells.

###  Scaffold Morphology and Pore Diameter

 Scaffold morphology was examined by SEM (JEOL JSM7100F) after sectioning, mounting on an aluminum stub with carbon tape. The sample images were taken at 50x and 250x magnification. Thus, pore size was analyzed using ImageJ at five replications. Energy-dispersive X-ray spectroscopy (EDS) was used on the same equipment for elemental analysis.

###  Porosity

 Dry composites were first weighed (W_0_), then fully submerged in 100% ethanol. After removal, residual ethanol on the outer layer was gently blotted, and the composites were weighed again (W_1_). Each group was tested in five replicates were evaluated to calculate means and standard deviations.^34^


$$Porosity\left(\%\right):\frac{W_{1}-W_{0}}{\rho \times V}\times 100\%$$

###  Swelling Ratio

 The scaffolds were initially submerged in PBS at 37°C (W0) and incubated for 1, 3, 6, 12, and 24 hours. After each time point, traces of PBS on the scaffold surface were thoroughly blotted before analysis before being analyzed.^34,35^


$$Swelling\;ratio\left(\%\right):\frac{W_{1}-W_{0}}{W_{0}}\times 100\%$$

###  Degradation Ratio

 The dry samples (W_0_) were initially soaked in PBS at 37°C PBS solution for 1, 2, 3, and 4 weeks. After incubation, the samples were removed and dried in an oven (W_1_).^28^ The degradation ratio was derived utilizing the following equation:


$$Degradation\;ratio\left(\%\right):\frac{W_{1}-W_{0}}{W_{0}}\times 100\%$$

###  Compression Strength

 A RT1-1225 A&D Company Universal Testing Machine (UTM) was used to evaluate the composites’ compression strength.^33^ The composites had rectangular shapes with a length-to-diameter ratio of 2:1 and were compressed with a pressure of 2.5 kN, as previously studied by Wang et al^30^ and Zhao et al.^36^


$$Compressive\;strength\left(MPa\right):\frac{Fc}{A}$$

###  Scaffold Bio Functionality Test

 MG-63 cells (5 x 10^5^) were propagated on a composite in a 24-well plate, which was previously coated with 5% Pluronic-127 for 30 minutes. After 6 h of adhesion, composites were replaced with new media on a new 24-well plate and cultured for 96 hours. Tetrazolium salt solution was added to the well to quantify cell viability, and the cells were cultured for an hour at 37 °C. The absorbance was determined at 450 nm with a Perkin Elmer multi-plate reader. For microscopy, samples were first fixed with 4% paraformaldehyde and then dehydrated in graded ethanol (70-100%).

## Results and Discussion

**Figure 1:**
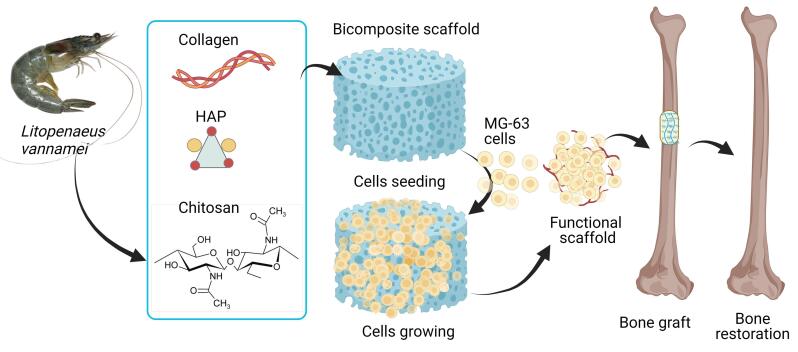
Scheme of Chitosan-based Scaffold from *Litopenaeus vanamei*Shell Waste for Bone Tissue Engineering. HAP: hydroxyapatite. This figure created using BioRender.

 The fabrication process of chitosan-based scaffolds is described in Figure 1. Shrimp shell-derived chitosan was combined with micro-fine HAP and COL I to engineer bio bio-composite scaffold, then the bio-functionality of the scaffold was assayed by seeding osteoblast-like cells (MG-63) into the scaffold. Such scaffolds are designed to support osteoblast growth *in vitro* and, in future applications, may be transferred to bone defects to facilitate regeneration.

 Chitosan extraction from Shrimp (*Litopenaeus vannamei*) shells.

**Figure 2:**
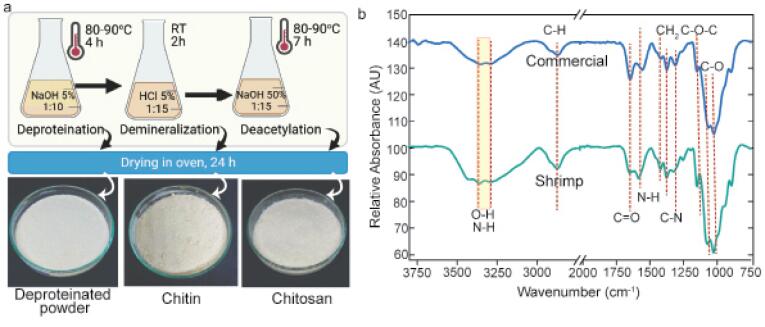
Chitosan extraction from *Litopenaeus vannamei* Shells. a. Steps of chitosan extraction and the results, b. Fourier Transform Infrared Spectroscopy (FTIR) spectroscopy analysis of extracted chitosan. RT: room temperature, NaOH: sodium hydroxide, and HCl: chloride acid

 This study extracted chitosan from shrimp (*Litopenaeus vannamei*) shells in three stages: deproteination, demineralization, and deacetylation (Figure 2a). Crustacean shells are primarily composed of calcium and magnesium carbonate (20- 50%), protein (20- 40%), chitin (15-40%), with minor constituents including lipid, astaxanthin, and other minerals.^37,38^ Proteins are removed from shells using 5% sodium hydroxide at 80°C for 4 hours. The weight lost during deproteination was considered as protein as much as 57.04 ± 8.73 %. During the demineralization step, the residual shell is exposed to 5 % hydrochloric acid, which removes the minerals through reactions with CaCO_3_ and Ca_3_(PO_4_)_2_. The powder obtained from this stage is chitin, which displays limited dissolvability and chemical activity because of its stiff structure and numerous hydrogen bonds. The powder obtained from this step is chitin, which is turned to chitosan by cleaving the acetamide group with concentrated sodium hydroxide, resulting in acetate ions and an -NH_2_ group that renders the chitosan acid-soluble.^38,39^ The chitosan extracted from shrimp shells was 22.37 ± 1.25%, appearing as an off-white powder. It is similar to previous studies by Ngerngyuang and colleagues^40^ but less white than the chitosan isolated from crab shells from our previous study. It is explained by the presence of higher astaxanthin in shrimp shells than crab shells, resulting in a more intense color of chitosan powder.^41^ Since the chitosan property is determined by the degree of deacetylation (DD), the DD determination was important to calculate DD before scaffold fabrication. The DD of shrimp shell chitosan was 77.22% while the commercially available chitosan was 69.87%, calculated based on Fatima.^32^ This result was consistent with previous studies in which *Litopenaeus vannamei* shell-derived chitosan has a DD value of about 80%.^40-42^ In the deacetylation step, the acetyl groups are removed from chitin molecules by a high concentration of NaOH to form an amino group (-NH_2_). The high degree of these amino groups is correlated with the reactivity of chitosan molecules.^43^

 To confirm the functional groups, FTIR spectra were used to compare extracted chitosan and commercially available chitosan as the standard, as revealed in Figure 2b. Both of the chitosan exhibited identical spectra, with a peak at 3363-3293 cm^−1^ corresponding to the stretching vibrations of water and hydroxyls, and free amino groups (NH_2_). The signal at 2875 cm^−1^ indicates an asymmetric stretching of CH_2_ in chitosan. The remaining acetamido groups stretch their −C−O bonds, resulting in amide frequencies of 1650 and 1557 cm^−1^. At 1419 cm^−1^, the spectrum exhibited −NH_2_ deformation, whereas the band at 1376 cm^−1^ represents C−C−H symmetric bending typical of alcohol groups. The C−N stretching was detected at 1311 cm^−1^, and the −CO stretching of alcohol groups was observed at 1068 and 1029 cm^−1^ for the alcohol groups’ vibration.^40^ The peak of C-O-C asymmetric stretching vibration appeared at 1150 cm^−1^, representing glycosidic bands.^44^ Polysaccharide fingerprints exhibit a distinct infrared spectrum in the 1000-920 cm^−1^ area due to variations in glycosidic link structure.^40^ In the FTIR spectrum, C-C and C-O stretching vibrations were detected in Region II (1200-800 cm^−1^), while Region V (3600-3050 cm^−1^) corresponded to OH stretching vibrations and Region IV (3050-2800 cm^−1^) to CH/CH_2_ stretching vibrations.^45^ Since the extracted chitosan has a band stretching pattern that matches the stretching band of commercial standard chitosan, it is confirmed that the isolated biomaterial is indeed chitosan.

**Figure 3:**
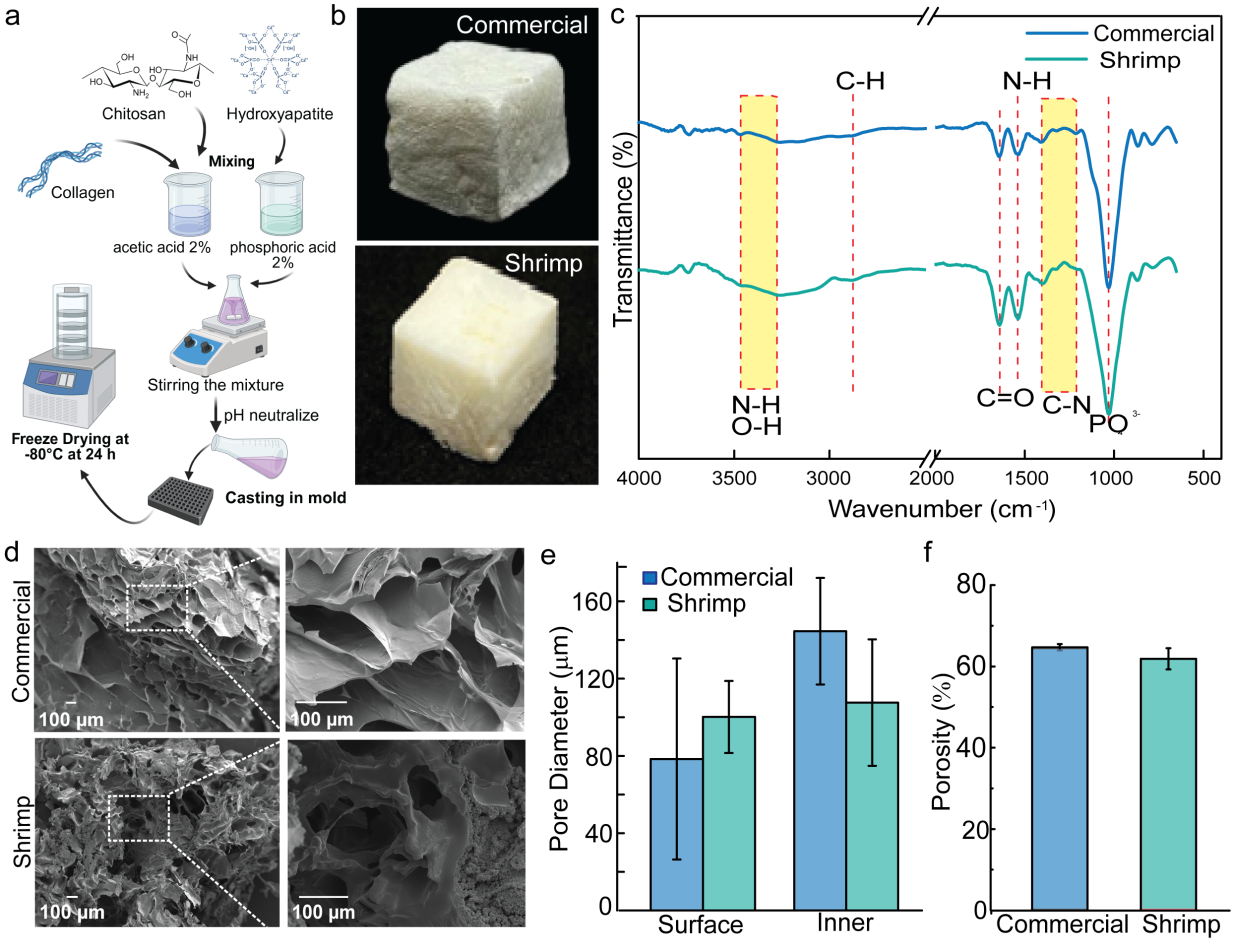
Fabrication, macroscopic, and microscopic characteristics of shrimp and commercial chitosan-based scaffolds; a. Fabrication scheme of the scaffold, b. Macroscopic image of the fabricated scaffold, c. FTIR spectra of engineered and commercial chitosan-based scaffolds, d. Scanning Electron Microscopy (SEM) images of the scaffold’s surface, e. Pore diameters of the surface and inner of engineered and commercial chitosan-based scaffolds (n = 5), f. The porosity of engineered and commercial chitosan-based scaffolds

 Furthermore, we engineered a scaffold using shrimp shell-derived chitosan incorporating COL I and HAP for osteoblast proliferation. The composite solution was then cast in a silicone mold and dried using the lyophilization method (Figure 3a). The engineered scaffold was an off-white and sponge-like 3D structure (Figure 3b). FTIR spectroscopy was employed to investigate the chemical composition of the composite scaffold. The broad band at 3500-3300 cm^−1^ represented N-H and O-H stretching vibration, which indicates intra- and intermolecular hydrogen bonds, typically associated with COL I, and chitosan structures.^43,46^ In addition, the presence of peaks at 1651, 1588 and 1416 -1250 cm^−1^ corresponded for C = O stretching, amide I, and C-N stretching vibration, confirming the characteristic functional groups of the scaffold component ^16,43,44^ N-H group bending is investigated to be responsible for a peak at 1537 cm^−1^.^45^ A recent study identified the absorption of the PO^4−^ group at 1095, 1031, 961, 603, and 563 cm^−1^.^18^ Our study discovered a distinct and strong peak at 1029 cm^−1^, which attributes to the PO4^3−^ group (Figure 3c). Hence, the peak for the hydrogen phosphate group appeared at 875 cm^−1^, and the carbonate groups appeared at 1407 cm^−1^.^46^

 Hence, collagen I specific bands such as amide A (3200-3300 cm^−1^), amide B (2950- 2919 cm^−1)^ could not be clearly observed due to overlapping bands with for N-H and O-H groups of chitosan.^47^ Moreover, further analysis is needed to accurately determine the elements’ composition of the scaffold using EDS. The presence of Ca and P was indicated by strong peaks for calcium and phosphate in the EDS spectra (Figure S1, Table S1). The shrimp shell scaffold was constructed of O (46.17%), Ca (21.68%), C (21.34%), and P (10.80%), which aligned with a previous study of Chi/Col I/HAP composite.^24^ To sum up, the lyophilization method has successfully engineered a biomimetic scaffold for bone tissue regeneration.

 Porosity and pore size are characteristics of the scaffold that are crucial for tissue regeneration. In tissue engineering, they are frequently tailored to meet the unique requirements of the desired tissue. Thus, we examined our scaffold microarchitecture under SEM. Both the shrimp shell and the commercial chitosan scaffold exhibited an open pore structure with irregular shape and an interconnected network (Figure 3d). The surface pore diameter of 78.38 ± 52.00 and 100.17 ± 18.65 µm; while the inner pore diameter was 144.5 ± 27.57 and 107.56 ± 32.32.72 µm, respectively, for commercial and shrimp shell chitosan scaffold (Figure 3e). The wide range of pore diameter is caused by the irregular pore shape of the scaffold. Thus, the porosity of the scaffolds was 61.85 ± 2.61% and 64.71 ± 0.77% for commercial and shrimp shell chitosan scaffolds (Figure 3f). Porosity percentages vary from 30% to 90% or higher, depending on many variables such as the tissue type undergoing regeneration, the desired mechanical properties, and the method used in scaffold development. The porosity of bone tissue varies from 70% to 90% in spongy bone and 5% to 30% in compact bone. In tissue engineering, porosity is often tailored for each application to balance features like stiffness and cell infiltration; thus, scaffolds have no established or typical value.^48^

**Figure 4:**
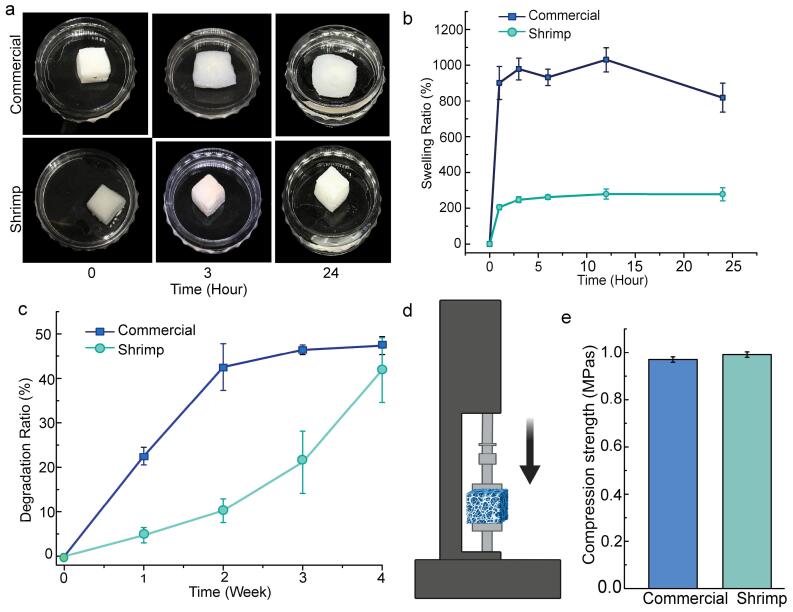
Swelling, degradation, and compression strength characteristics of shrimp and commercial chitosan-based scaffolds; a. Swelling images of the scaffolds at 0, 4, and 24 hours; b. Swelling ratio of the scaffolds at 0, 1, 4, 6, 12, and 24 hours; b. Degradation images of the scaffold at 0, 2, and 4 weeks (n = 5); d. The degradation ratio of the scaffolds 0-, 1-, 2-, and 4-week (n = 5), e. Scheme of compression strength test, f. Compression strength of shrimp and commercial chitosan-based scaffolds (n = 3)

 A crucial factor for a scaffolding system in tissue engineering is water uptake. Controlling swelling behavior is similarly important to managing porosity when it involves maintaining the scaffolds’ fidelity and integrity during interactions with biological fluids. This characteristic influences the transport of nutrients into the scaffold so that they support cell availability, proliferation, and differentiation. The scaffolds became swollen in PBS (pH 7.4) at 37 °C for 24 hours to determine the amount of water that the scaffold absorbed (Figure 4a and 4b). The swelling ratios of the commercial scaffolds were 900.97 ± 91.77, 978.69 ± 60.38, 1030.38 ± 67.86, 818.56 ± 81.03, and 931.76 ± 45.64 % at 1, 3-, 6-, 12-, and 24-hour incubation. While shrimp shells scaffolds had a swelling ratio of 204.79 ± 13.53, 247.56 ± 16.94, 262.64 ± 12.50, 279.44 ± 28.02, and 278.55 ± 36.49% at the same duration of incubation. Because chitosan and COL I are both hydrophilic materials, the designed scaffolds were demonstrated to have a significant swelling ability.^49^ Chitosan in the scaffold composite with other polymers, such as polyvinyl alcohol (PVA), was reported to increase the swelling ratio.^50^ The water uptake capacity of commercial chitosan scaffolds was drastically higher than shrimp shell chitosan scaffolds. It is considered that commercially available chitosan takes up take water than the shrimp shell chitosan. Since both scaffolds displayed no significant difference in porosity and pore diameter, the existence of impurities such as calcium carbonate and protein hinders the water uptake properties of the shrimp shell chitosan.^51^

 In tissue engineering applications, biodegradability is a crucial characteristic for building scaffolds. Ideally, the scaffolds will break down through a regulated process and become integrated by the surrounding tissues without requiring surgical revision.^52^ In addition, lysozyme catalyzes the breakdown of chitosan over its β-1–4-glycosidic bonds.^53^ The rate at which porous scaffolds degrade affects cell vitality, cell proliferation, and even host responses. The degradation ratio of the commercial chitosan scaffold was 22.53 ± 1.99, 42.56 ± 5.26, 46.43 ± 1.11, and 47.37 ± 2.03% at 1, 2, 3, and 4 weeks. While shrimp shells chitosan had degradation ratios of 4.74 ± 1.72, 10.25 ± 2.67, 21.15 ± 17.00, and 41.87 ± 7.27%, respectively. This reduced degradation could be attributed to its lower purity of the isolated chitosan, which makes it slower to absorb water, thereby delaying degradation. Previous studies also reported that chitosan extracted from crustacean shells exhibited slower degradation rates due to its heterogeneous structure, residual minerals, lower accessibility to hydrolyzed enzyme and variable DD, compared to highly purified commercial chitosan.^54,55^

 Designing composite scaffolds for bone tissue engineering requires a thorough investigation of their mechanical properties. Bone strength and load-bearing capacity are the two major components of bone healing, depending on the scaffolds’ mechanical strength. The mechanical strength of scaffolds is a crucial property for the integrity as well as proliferation of the osteoblast.^53^ During compression testing works by putting the scaffold n a solid stage and pressed from above to evaluate its resistance to compression (Figure 4e). The shrimp shell scaffolds had a compressive strength of 0.991 ± 0.01 MPa, compared to 0.97 ± 0.01 MPa for the control scaffold (Figure 4e). All quantitative data were provided as SD for three samples. Although the scaffolds exhibited lower compressive strength than cortical bone (100- 230 MPa), their values fell within the range of cancellous bone (2-12 MPa), suggesting suitability for trabecular bone applications. Even though their mechanical strength did not meet the criteria for bone tissue engineering, chitosan-based scaffolds seeded with osteoblast or stem cells had increased mechanical properties due to matrix mineralization.^56,57^

**Figure 5:**
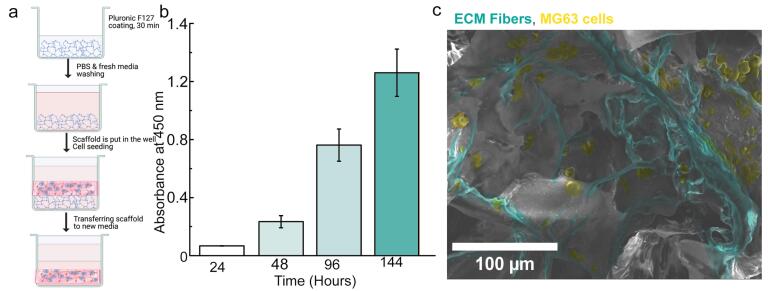
Biocompatibility test of engineered scaffolds: a. Scheme of cell seeding on the scaffold, b. The absorbance of formazan complex at 450 nm after 24-, 48-, and 144-hours incubation (n=4), c. SEM image of MG-63 cells and the extracellular matrix fibers on the scaffold (yellow: MG-63 cells, green: ECM fibers). PBS: phosphate buffer saline.

 An ideal tissue engineering scaffold ought to be attached by the cells and not trigger cell cytotoxicity, which is assessed using cell binding and *in vitro* MTT assays. The cell binding protocols were conducted based on our previous studies, which were slightly modified by seeding the cells on the scaffold in the Pluronic-coated well plate (Figure 5a).^14,15^ The viability of the cells was measured by tetrazolium salt absorbance at 450 nm. On 24, 48, 96, and 144 hours after cell seeding, the absorbance was 0.112 ± 0.01, 0.237 ± 0.09, 0.765 ± 0.201, and 1.317 ± 0.254, respectively (Figure 5b). The MG-63 cells’ attachment and support were supported by COL I in the fabricated scaffold, which was considered the main factor instead of the interconnected network of the chitosan scaffold.^23^ Moreover, the cells’ morphology on the scaffold was observed under SEM microscopy 4 days after seeding (Figure 5c). Microarchitecture of an open connected pore in the scaffold due to the interaction with the cells. The cells bind to the scaffold on the RGD peptide of collagen through the integrin receptor, then they secrete the ECM network surrounding the cells and remodel the interconnected networks of the scaffolds^58,59^ as Figure 5c. Thus, this interaction between cells and scaffolds happened reciprocally, giving biochemical cues for cell proliferation to be a specific tissue. Besides its microstructure, the chemical properties of the scaffold affect the behavior of cells to determine the cell’s fate.^60^

 Taken together, this study developed a biomimetic scaffold for bone tissue engineering incorporating chitosan derived from the shrimp *Litopenaeus vannamei* shells with COL I and HAP. It gives an insight into the potential use of marine biowaste for the fabrication of biopolymers by comparing the physicochemical properties, degradation behavior, and biological performance of shrimp shell-derived chitosan scaffolds and commercial chitosan scaffolds. The detailed characterization and *in vitro* analysis of our engineered scaffolds will elucidate their prospects for bone regeneration, especially for the development of cost-efficient strategies and greener materials for use in biomedicine.

## Conclusion

 This study successfully engineered shrimp shell-derived chitosan to be a biomimetic scaffold that supported osteoblast viability. The extracted chitosan had a high DD and sufficient purity for scaffold engineering, and its chemical characteristics were similar to those of commercial chitosan. Chitosan and HAP’s distinctive peaks were easily distinguished in FTIR spectra, although overlapping peaks covered up collagen-specific bands. Because of their higher purity, commercial chitosan scaffolds showed a higher swelling ratio and degradation than the engineered scaffolds. In the range of cancellous bone, both scaffold types demonstrated comparable compressive strengths and promoted the adhesion and proliferation of MG-63 cells, demonstrating biocompatibility. These results demonstrate that chitosan made from marine biowaste has the potential to be an economical and environmentally friendly substitute for bone tissue engineering applications.

## Competing Interests

 There is no potential conflict of interest was reported by the authors.

## Ethical Approval

 Ethical approval was obtained from the Ethics Committee of the Faculty of Health Scinces, Respati University, Yogyakarta with approval number 051/FIKES/PL/III/2023.

## Supplementary File

 Figure S1. Energy dispersive spectroscopy on the scanning electron microscope (EDS-SEM) of fabricated scaffold.
Table S1. Result of energy dispersive spectroscopy (EDS) spectrum analysis.
