# Supplementary material for: Biomimetic Scaffold of Chitosan from Litopenaeus vannamei Shrimp Shells Incorporated with Collagen and Hydroxyapatite for Bone Tissue Regeneration
Source: Adv Pharm Bull. 2025 Dec 21;16(1):52–61. doi: 10.34172/apb.025.45909 (PMC13408001; doi:10.34172/apb.025.45909)
Supplement: Supplementary file 1 — Figure S1. Energy dispersive spectroscopy on the scanning electron microscope (EDS-SEM) of fabricated scaffold. Table S1. Result of energy dispersive spectroscopy (EDS) spectrum analysis. [file apb-16-52-s001.pdf]

Adv Pharm Bull 2026;16(1):52-S1
doi:10.34172/apb.025.45909
https://apb.tbzmed.ac.ir

Supplementary

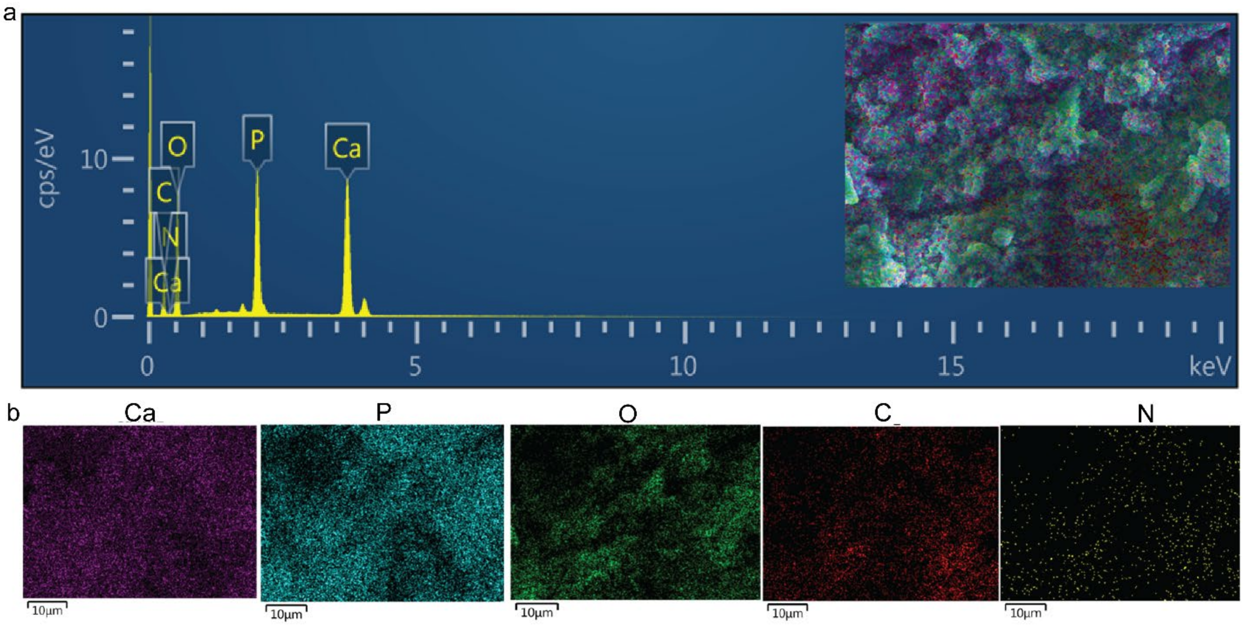

Figure S1. Energy dispersive X-ray spectroscopy (EDS) spectra and elemental mapping of *Litopanaeus vanamei* shell derived-chitosan scaffold. a. EDS analysis of the scaffold, b. Elemental mapping of C, N, O, P, and Ca of the scaffold.

Table SI. Element Analysis using EDS

Element	Weight Percentage
C	21.34
N	0.00
O	46.17
P	10.80
Ca	21.68
